# A Skeptic’s Testimony

**Published:** 1889-09

**Authors:** 


### A SKEPTIC'S TESTIMONY.

We re-publish from the *Banner of Light*, edited for so many years by our venerable friend Luther Colby, the following remarkable statement of the writer's experiences at a public meeting, presided over, as we have ascertained, by Mrs. Ada Foy, of San Francisco, Cal., one of the most gifted sensitives of whom we have any knowledge. We give it with the assurance that the whole narrative is strictly true, having been accorded an interview with the writer, notwithstanding his expressed repugnance to it. ED.

The following narration of facts may be relied upon as true in all particulars. The writer is a practicing lawyer, having an office contiguous to mine, and at my request reduced them to writing. It seems to me not a little singular that one so clear-headed and self-reliant as my friend is known to be, should feel called upon not only to disavow any belief in a species of phenomenon of the truth of which his personal experience furnishes a remarkable example, but so willfully bar the door again further investigation. NELSON CROSS.

No.—Broadway, N. Y., May 27, 1889.

DEAR SIR: At your request I write out what to me was a remarkable occurance, and concerning which there cannot enter the element of uncertainty.

To understand the matter thoroughly I will give a short introduction. You know that I am a lawyer in active practice in this city, with all the conservatism of my profession, a disbeliever in all spiritual phenomena, and at an age when one's mental powers are presumed to be in their prime.

A number of years ago I formed the acquaintance of a lawyer who occupied an adjoining office, a man much older than I, S——— by name, and between us sprung up a very close friendship. He was a cool, wary, shrewd man, of a daring and philosophic turn of mind, a disbeliever in all systems of religion, carrying his disbelief to the extent of denying the immortality of the soul and the existence of a God.

He was truthful, honest, fearless, and lived and died without a blemish on his name—the possessor of a fortune accomplished by his own efforts.

We were accustomed to argue constantly on all subjects of current interest, and frequently would attempt the solution of problems in the realms of mental science.

On one question, the immortality of the soul, we radically differed, and many an argument we had thereon, till one day we made the following agreement, which was known but to him and me, viz : That the one who should first die should inform the survivor of the simple fact whether he was living after what we called death.

My friend died about a year ago, and having the agreement in mind, I attended one or two Spiritualist meetings as an experiment, and not with the slightest idea of receiving any communication, for I was and am a skeptic of the skeptics as to any, every and all spiritualistic doctrines, teachings and phenomena.

On Tuesday May 21, 1889, I received an invitation from a client to attend a meeting ; out of curiosity I accepted, and on my way told the gentleman who invited me the story of myself and friend, and laughingly remarked that I would call up S——.

We arrived late ; the hall was crowded and brilliantly lighted ; we took our seats at the rear, well to one side, and almost screened from observation.

After the lecture, the speaker, whom I never saw or heard of before, invited those who wished to communicate with some departed friend to write the name on a slip of paper, fold it securely so as to hide the name, and put it in the hat which would be passed around. I did so, wrote my friend's name on a slip I tore from a blank check, placed it in the hat, with, I suppose, a hundred others, and saw the hat placed upon the table.

By this time the only sentiment that moved me was a kind of scornful curiosity, a pity for what I considered sentiment "run mad," and a sort of impression that the audience were about to be cleverly humbugged.

After perhaps a half an hour had passed, and a dozen more or less communications had been received, which to my mind were very unsatisfactory—because either through the thoughtlessness of the parties sending or receiving the messages there seemed to me to be an unlimited opportunity for fraud or nonidentification—the medium took up the slip unopened that I had sent up, said : "There is a spirit here, S——— by name, who says that he has come to communicate with one who has long been anxious to see him."

I then rose, and said, "Madam, I think that must be for me. Will you ask the gentleman for his Christian name?" She answered, "Edward." The unopened slip meanwhile was delivered to a stranger in the audience. I will now go on and give you the colloquy. Mind you, I was not in the slightest degree nervous; I was cool and skeptical as when cross-examining a witness on the stand; and in fact, for the time being my professional instincts got the better of me, and I framed my questions accordingly. I now take up the questions. I spoke to him by name as I would to a witness:

QUES. What was the middle letter of your name? ANS. H.

Q. What was your business in life? A. Lawyer.

Q. What city did you practice in? A. New York.

Q. On what street when I first knew you? A. Broadway.

Q. What number? A. 73.

Said I, "You are wrong." The medium hesitated a second, appeared to reflect, then replied.

"The spirit says that *he* is right, and you are wrong; you were in 71; he was in 73."

This is true, but I had forgotten the fact; it was all one building, and he was on the opposite side of the hall, in No. 73, while I was in No. 71.

Q. Where did you die? A. Plainfield, New Jersey.

Q. Did you have a corporation for a client on Chambers Street? A. Yes.

Q. Name it? A. American News Co.

Q. What kind of a suit did you have for them in which I helped you? A. Libel suit.

Q. What was the result of the trial? [No answer.]

Q. What was then done? A. Appealed.

Q. Where to? A. General term.

Q. What court? A. Superior Court.

Q. What result? A. Judgment reversed.

Q. What then was done? A. Appealed to Court of Appeals.

Q. What was the state of the action at the time of your death? A. Appeal pending.

Q. Give plaintiff's name? A. Marie Prescott.

Q. What was your belief when you died? A. I believed in none of the systems of relgion. I went further; I disbelieved in the immortality of the soul.

Q. S———, how did you come to attend here to-night? A. I came to redeem the agreeement you and I made at No. 71 Broadway, that the one who first died should inform the survivor whether or no he lived.

I confess at this point I was startled and felt that either my mind was an open book to the medium, or else I had called upon "the devil;" and I put but one more question. Said I:

Q. S———, what are you doing? A. I am studying, learning, teaching, and sometimes I assist you; good night!

This was all. I knew no one except the gentleman who attended with me, and he was as great a skeptic as myself. He never left my side! I was at a distance of fully forty feet from the medium, and between her and my-self must have sat fifteen or twenty persons, and within a less radius fully three hundred. As I said before, I never saw her, and never told any one of my agreement save the gentleman who was with me, and him that night on our way to the meeting.

I have no theory on which to account for it. As I have stated to you, and as you well know, I am a person of strong will. I have never seen any one able to mesmerize me. I am skilled in trying cases, and in concealing from witnesses and litigants my thoughts, and have never yet seen any one who could do more than guess at the working of my mind.

I leave to wiser heads than mine the solution of this incident. I shall never again call upon the spirit of my friend, or give anyone the chance of reading my thoughts, whichever way it may be decided, and I write this out at your request upon the express agreement that my name is not in any way to be used nor published, nor am I to be bothered with the queries of any one concerning the transaction.

I remain as ever,

Yours very sincerely.

